# The Chemical Composition and Metabolic Effects of *Attalea phalerata* Nut Oil in Hyperlipidemic Rats Induced by a High-Fructose Diet

**DOI:** 10.3390/molecules23040960

**Published:** 2018-04-20

**Authors:** Débora da Silva Baldivia, Eliana Janet Sanjinez-Argandonã, Kátia Ávila Antunes, Izabel Cristina Freitas Moraes, Edson Lucas dos Santos, Kely de Picoli Souza

**Affiliations:** 1 Research group on Biotechnology and Bioprospecting applied to metabolism (GEBBAM), Federal University of Grande Dourados, Rodovia Dourados-Itahum, Km 12, Dourados MS 79804-970, Brazil; baldivia_bio@hotmail.com (D.d.S.B.); avilacatia@yahoo.com.br (K.A.A.); edsonsantosphd@gmail.com (E.L.d.S.); 2 College of Food Engineering, Federal University of Grande Dourados, Dourados MS 79804-970, Brazil; eliana.argandona@ufgd.edu.br; 3 Department of Food Engineering, University of São Paulo, Pirassununga SP 13635-900, Brazil; bel@usp.br

**Keywords:** bacuri, fatty acid, body weight, visceral adiposity, hypercholesterolemia

## Abstract

The fatty acids found in nuts are important regulators of the metabolism. These acids are frequently associated with a reduction of serum cholesterol and body fat and a lower risk of developing cardiovascular disease. In this context, the aim of this study was to identify and quantify the nut oil fatty acids from *Attalea phalerata* and investigate their metabolic effects in rats with hyperlipidemia induced by a diet rich in fructose. Oleic and lauric acids were the major compounds found in the *A. phalerata* nut oil (APNO). Hyperlipidemic rats treated with APNO showed a reduction in the total serum cholesterol similar to those treated with simvastatin, an increased body temperature by 1 °C, and a reduction in the body weight gain and mesenteric depot of white adipose tissue compared to the hyperlipidemic controls rats. There was an increase in the relative liver weight of rats treated with APNO, without, however, any change in the serum markers of hepatic toxicity. In addition, there was an increase in the moisture and lipid content of the feces of the rats treated with APNO compared to the controls. Together, these results suggest that APNO has potential use in health foods and nutritional supplements to control hypercholesterolemia and obesity.

## 1. Introduction

*Attalea phalerata Mart. ex Spreng* (Arecaceae) is a palm tree found in the central highlands of Bolivia, Brazil, Paraguay, and Peru [1], popularly known as the “bacuri,” and has economic importance as a source of vegetable oil [2]. This palm has bunches of fruit that have a fibrous, juicy pulp, a woody endocarp, and 2–4 hard nuts, which are mainly consumed fresh.

Consumption of the nuts is related to beneficial metabolic effects, such as the reduction of serum cholesterol and body fat [3], resulting in a lower risk of developing cardiovascular disease [4]. These effects are mainly related to the fatty acids found in the nuts [5], which are important sources of energy and precursors of bioactive molecules with different metabolic and biochemical functions [6].

Saturated fatty acids (SAFAs), the main constituent of coconut oil [7], are recommended in diets for weight loss because they are easily oxidized in the liver, increase energy expenditure, resulting in decreased corporal adiposity [8].

The consumption of monounsaturated fatty acids (MUFAs) is associated with the maintenance of body weight and a reduction in central adiposity, resulting in a lower risk of developing obesity [9]. Of the MUFAs, oleic acid is the major fatty acid found in olive oil, the main component of the Mediterranean diet, which has rapid absorption in the body and shows a hypocholesterolemic effect [10]. Like the MUFAs, the polyunsaturated fatty acids (PUFAs) also lower low-density lipoprotein cholesterol (LDL-C) and promote the stabilization of atherosclerotic plaque [11].

Although the consumption of fatty acids is described according to their beneficial effects on the health of the consumer, scientific information is still limited and controversial regarding the side effects of a prolonged intake of fatty acids of vegetable origin and there are no scientific reports on the effects of the prolonged consumption of *A. phalerata* nut oil. Therefore, this study identifies and quantifies the fatty acids present in *A. phalerata* nut oil for the first time and investigates its metabolic effects on rats with diet-induced hyperlipidemia.

## 2. Results and Discussion

### 2.1. Yield and Composition of the Fatty Acids in A. phalerata Nut Oil (APNO)

The yield of nut oil extracted from APNO was 29.91%. Mechanical pressing results in partial removal of the nut oil, with lower oil retrieval when compared to the extraction with organic solvents. However, in terms of food safety, the oil extracted by pressing is of better quality than the solvent-extracted oil, due to the absence of chemical residues in both the oil and the flour, a byproduct generated after oil extraction that can be used for food for both humans and animals.

The average value obtained for the acidity of the APNO after extraction was 0.12%, within the limits observed for oils according to *Codex Alimentarius* [12].

The chemical composition of APNO (Table 1) showed higher concentrations of saturated fatty acids (28.9% lauric, 12.2% myristic, and 10.7% palmitic acid), followed by monounsaturated (30.7% oleic acid) and polyunsaturated (4.75% linoleic acid). The other fatty acids found in APNO showed concentrations below 4.5%.

The growing interest in fatty acid-rich foods is due to the health benefits they provide. Oleic acid, the major compound of APNO, is an important monounsaturated fatty acid that is rapidly absorbed, supplies energy, and reduces body mass [13]. Lauric acid is highly oxidized in the body [14]. It is inserted into the class of medium-chain fatty acids (MCFA), which are characterized by increasing energy expenditure, oxidizing fat, and reducing the deposits and body mass [15].

On the other hand, myristic and palmitic fatty acids are characterized by their atherogenic and thrombogenic activities [10]. Moreover, Voon, Ng, Lee, and Nesaretnam [16] reported that these fatty acids do not change the parameters related to cardiovascular risk. Thus, the mechanisms and biological responses of these saturated fatty acids are controversial and need to be better investigated.

Linoleic acid, present in APNO (Table 1) is described as hypocholesterolemic and reduces the risk of cardiovascular disease [17,18]. It is an essential fatty acid since it cannot be synthesized by the body [10], and thus, it is indispensable to obtain it from natural food sources. According to the Food and Agriculture Organization (FAO) [19], the daily intake of linoleic acid should range between 2.5–9%, and thus, APNO could be an alternative source of linoleic acid in the daily dietary recommendations. Of the polyunsaturated class of fatty acids, α-linolenic acid (18:3 n-3) is easily found in foods of animal and plant origin, but in limited quantities [20]. It regulates the oxidation of fatty acids by inhibiting lipid synthesis [21], thus reducing the risk of cardiovascular disease [22].

### 2.2. The Effects of A. phalerata Nut Oil on the Parameters of Food Intake and Feces and Urine Excretion

The administration of APNO did not change the food intake of the groups evaluated but reduced the water consumption in the high-fructose diet (HFD) + APNO group as compared to the HFD and control group (Table 2).

Although this change was not observed in the metabolic cages after 24 h, a lower urinary excretion was observed in the HFD + APNO group as compared to the control group (Table 3). The ratio of excreted urine to water intake during 24 h showed there was a 39% reduction in the water removal from the rats in the HFD + APNO group, indicating that although the water intake was lower, the water balance was preserved.

The feces of the rats treated with APNO were lighter in color, larger, and of a softer texture compared to the feces of the control rats. These characteristics may be attributed to the higher water and lipids content of the feces of these rats (Table 3).

The presence of water promoted the softening of the stools, facilitating intestinal emptying, similar to the effect of insoluble fiber [23]. However, the fecal dry weight of the rats in the HFD + APNO group was lower than that of the control group (51 versus 76%, respectively), indicating that the rats treated with APNO absorbed larger amounts of nutrients from the high-fructose diet.

### 2.3. Effect of A. phalerata Nut Oil on Body Mass, Temperature, and White Adipose Tissue Depots

**Figure 1:**
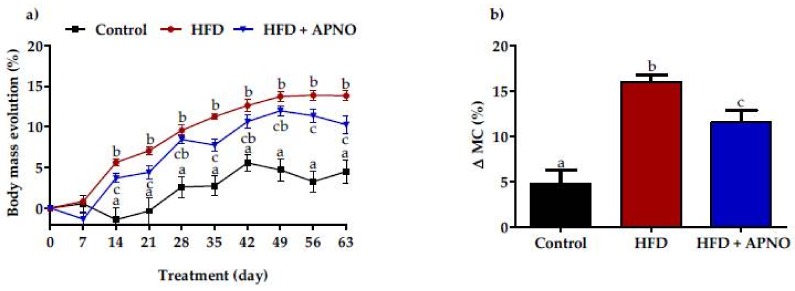
(**a**) Body mass evolution and (**b**) total body weight gain of normolipidemic rats (Control) and high-fructose diet-induced hyperlipidemic rats treated with water (HFD) or *A. phalerata* nut oil (1.2 mg·Kg^–1^ of body weight, HFD + APNO) for 63 days. Results are expressed as the mean ± SEM. *n* = 8 animals per group. The data was analyzed by one-way ANOVA test followed by a Student Newman–Keuls post-test. Different superscript letters indicate significant differences at *P* < 0.05.

Hyperlipidemia and obesity, induced by high-fructose diets in experimental models, are well described in the literature [24,25,26]. In the present study, the rats treated with the high-fructose diet (66% fructose) showed greater body mass gain when compared to rats fed the commercial rodent diet (control group) (Figure 1a,b).

The rats receiving *A. phalerata* nut oil (HFD + APNO) showed reduced body weight gain as compared to the HFD group (Figure 1b), accompanied by a decrease in mesenteric adipose depots (Table 2), one of the main deposits related to inflammation in obesity, which contributes to increased cardiovascular risk [27]. Corroborating these results, Poudyal et al. [28] showed that a diet rich in fructose supplemented with oils containing the oleic, linoleic, and α-linolenic fatty acids was capable of reducing body fat in rats. In addition, Assunção et al. [29] showed that the intake of coconut oil, which is rich in lauric acid, promotes the reduction of abdominal fat in obese women. Thus, the effect of APNO in reducing adiposity may be related to the presence of the oleic, linoleic, α-linolenic, and lauric fatty acids, considering their ability to alter the lipid metabolism and reduce the fat content.

Another factor that may have contributed to the reduced evolution of body weight and reduction in mesenteric adipose tissue in rats in the HFD + APNO group was the increase in body temperature (Table 3), directly related to the effect of increased energy expenditure. This result is in agreement with reports by Noguchi et al. [30] and St-Onge and Jones [8], describing the intake of vegetable oils rich in medium chain fatty acids (MCFAs) and long-chain fatty acids (LCFAs) increases thermogenesis and energy expenditure, and decreasing the accumulation of fat deposits, thus, showing a reduction in body mass.

### 2.4. Effect of A. phalerata Nut Oil on the Mass of Muscle Tissue and Organs

The relative mass of skeletal muscles (extensor digitorum longus and soleus) and organs (spleen, heart, lung, and kidney) showed no differences between the groups, but the liver of the HFD + APNO group showed a statistical difference as compared to the HFD group (Table 2).

The liver mass of the HFD + APNO group was higher when compared to the control and HFD groups (Table 2). However, the percentages of lipids in the liver of the rats in the HFD + APNO and control groups were similar but were lower in the HFD group (Table 2). In addition, no changes were observed in the serum levels of the marker enzymes of hepatotoxicity, aspartate aminotransferase (AST), and alanine aminotransferase (ALT) (Table 4). According to Edem [31], an excessive consumption of SAFAs can injure the hepatocytes, releasing large amounts of the alanine aminotransferase (ALT) and aspartate aminotransferase (AST) enzymes in the bloodstream, which indicates their toxicity. In the present study, neither the liver enzymes AST and ALT nor the serum creatinine was changed by the treatment with APNO as compared to the HFD controls (Table 4), indicating that up to the assessed dose, there were no toxic effects, according to the parameters.

Previous studies have shown that oral administration at doses ranging from 125 to 2000 mg·kg^−1^ of the pulp oil of *A. phalerata* rats did not show any signs of toxicity and that the oil was considered safe in the doses evaluated [32,33]. According to our results, the intake of APNO (30.68% oleic acid, MUFA) can be considered safe for hepatic health, since studies have shown that MUFA-rich olive oil is considered a source lipid function that best preserves the liver during the aging process [34] and recovers the liver with hepatic steatosis [35]. The longer use of APNO (over 63 days) needs to be better investigated to follow the hepatic impairments observed.

Treatment of the rats with ciprofibrate (HFD + C) and simvastatin (HFD + S) resulted in a reduction in serum triglycerides, an effect not seen in the group that received the APNO (Table 4). Although the APNO showed a high concentration of oleic acid (30.9%), the serum triglyceride levels of the HFD + APNO group were similar to those of the HFD group (Table 4). This result is in agreement with the observations made by Poudyal et al. [28] and contrary to those made by Kris-Etherton et al. [36], relating to the intake of food rich in MUFAs and the reduction triglycerides.

The treatment with APNO reduced the serum cholesterol levels of the rats in the HFD + APNO group similar to the reduction in the HFD + S (simvastatin) and HFD + C (ciprofibrate) groups, as compared to the HFD group (Table 4). Statins and fibrates are drugs used clinically to reduce the serum cholesterol lipids and triglycerides in humans [37,38]. Statins inhibit HMG-CoA reductase, which promotes the reduction of cholesterol synthesis in liver cells, whereas the fibrates act as PPAR-α agonists that lead to the further degradation and excretion of lipids [38].

The hypocholesterolemic activity of APNO can be related to the concentrations of MUFAs (30.74%) since a reduction in serum cholesterol levels is amongst the major beneficial effects of the intake of this class of fatty acids [32]. This effect was also observed in rats [28] and humans [11] fed diets rich in oleic (MUFA), linoleic, and α-linolenic (PUFAs) acids. The chemical constituents of the APNO are probably responsible for the hypocholesterolemic activity observed.

Additionally, the hypocholesterolemic effect of APNO, added to the observed increase in temperature, may be related to brown adipose tissue activation, leading to serum cholesterol reduction [39], promotes clearance of high-density lipoprotein cholesterol (HDL-C), increases the reverse transport of cholesterol from macrophages to feces and the hepatic elimination of HDL cholesterol [40].

## 3. Materials and Methods

### 3.1. Botanical Material

Mature fruit with a uniform appearance were collected directly from the ground under the canopy of *A. phalerata* trees in the city of Bonito, Mato Grosso do Sul state, Brazil (Latitude 21°10′19.1, longitude 056°26′. 58.0, 6 m altitude), with the permission of the governmental ‘Authorization and Information Biodiversity System’—SISBIO, n° 37931-2. The botanical material was identified and a voucher specimen was deposited in the herbarium DDMS/UFGD under n° 5033. The fruits were sanitized with 0.66% dehydrated sodium dichloroisocyanurate solution (3% active chlorine content) for 10 min, and the endocarp was broken to remove the nuts.

### 3.2. Extraction of the Nut Oil from A. phalerata

The nuts were dehydrated at 40 °C for 72 h in a convective dryer with an air flow of 0.5 m·s^−1^ and subsequently ground in a semi-industrial blender to a uniform mass. The cold-pressed oil was obtained from 300 g of this nut mass using an adapted screw press. After extraction, the *A. phalerata* nut oil (APNO) was centrifuged at 1100 rpm for 5 min to remove residues and the supernatant collected and stored in a brown bottle at 4 °C.

The APNO extraction yield was calculated from the difference between the initial mass and the mass of dried nut obtained at the end of pressing. After the extraction of the APNO, the acid value was determined by titration with a 0.1 N sodium hydroxide solution as determined by Standard AOAC (Association of Official Analytical Chemists) methods [41].

### 3.3. Chemical Composition of APNO

The fatty acid profile of APNO was analyzed on a gas chromatograph, model 3900 (Varian, Palo Alto, CA, USA), equipped with a split injection port, CP-Sil 88 capillary column (100 m × 0.25 mm id, 0.2 µm thickness), and a flame ionization detector (FID). The fatty acids were prepared by transmethylation, as described in [42], using an ammonium chloride solution and sulfuric acid in methanol as the esterifying agent. The operating conditions of the gas chromatograph (GC) were as follows: 270 °C injector temperature, 310 °C detector temperature, oven temperature programming ramp: 140 °C for 2 min, heating from 140 to 235 °C (2.5 °C·min^−1^) and remaining at 235 °C for 10 min. The injections were performed using hydrogen as the carrier gas at 13.7 psi. Fatty acid peaks were identified by comparison with retention times of fatty acid methyl ester standards (Sigma-Aldrich, Bellefont, PA, USA) (Supelco^®^ 37 Component FAME Mix Bell); Linolenic acid methyl ester (C18: 3 w3), Cis-7,10,13,16,19-docosapentaenoic acid methyl ester (C22: 5 w3), and Conjugated linoleic acid methyl ester (CLA), and the quantification was performed by normalized areas. The results were expressed as g·100 g^−1^ of the total fatty acids.

### 3.4. Diet-Induced Hyperlipidemia

A high-fructose diet (HFD) was prepared by mixing 34% of a commercial rodent diet (Presence) with 66% fructose. Both diets (HFD and commercial chow) were analyzed as follows: the lipid levels by the Soxhlet method; the fixed mineral residue by incineration in a muffle furnace at 550 °C; protein content by the Kjeldahl method; and moisture content by removing the water in the air circulating oven (105 °C) until a constant weight, according to the AOAC [41]. The carbohydrate content was estimated by the difference from the values obtained for the proximal composition [43]. Table 5 shows the proximal compositions of the commercial and high-fructose diets.

### 3.5. Animals

Wistar rats (*n* = 57) were used in this study, obtained from the Central Animal Facility of the State University of Maringa—UEM, Brazil—each weighing approximately 150 g. The rats were kept in polyacrylic cages in a well-aerated environment with a controlled temperature of 22 ± 2.0 °C, photoperiod of 12 h, with free access to food and water throughout the experiment. All the rats were maintained for 15 days of acclimatization prior to any experimental procedure.

The experimental procedure was carried out according to the standards of the National Council of Animal Experimentation—CONCEA—and approved by the Animal Ethics Committee of the Federal University of Grande Dourados—UFGD (Protocol n° 023/2012—CEUA/UFGD).

### 3.6. Experimental Design

#### 3.6.1. APNO Dose Determination

For the experimental determination of the APNO dose, the rats were fed the commercial diet and divided into 4 groups (*n* = 5). The control group was gavage fed water and the APNO groups were gavage fed APNO at concentrations of 0.6, 1.2, and 1.8 mL·kg^−1^ of body weight (BW), every six days, sequentially. The dose of choice was 1.2 mL·kg^−1^ of BW since this was the largest dose that did not cause any changes in the water intake, diet, weight change, feces consistency, irritability, piloerection, and hair loss.

#### 3.6.2. Hyperlipidemia Induced by the High-Fructose Diet

Before the experimental period, the rats were fed for 112 days on the commercial diet (*n* = 5, Control group) or the high-fructose diet (*n* = 32, HFD) to induce hyperlipidemia. The rats were subsequently divided into five groups: (I) commercial diet + water (Control group), (II) high-fructose diet + water (HFD group), (III) high-fructose diet + 2 mg·kg^−1^ of BW ciprofibrate (HFD + C group), (IV) high-fructose diet + 30 mg·kg^−1^ of BW simvastatin (HFD + S group), and (V) high-fructose diet + 1.2 mL·kg^−1^ of BW nut oil *A. phalerata* (HFD + APNO group). The treatments were daily by gavage for 63 days. Throughout the trial period, the food and water intake, as well as the evolution of body weight, were monitored three times a week.

#### 3.6.3. Evaluation of the Metabolic Parameters in Rats

After 60 days of treatment, the rats from the HFD and HFD + APNO groups were weighed and allocated individually to metabolic cages for 24 h for individual evaluation of food and water intake, and urine and feces excretion. At the same time, the anal temperature of the rats was determined with the aid of a digital thermometer (TS-101, Techline, Changchun, China) to evaluate the thermogenic effect of the APNO.

#### 3.6.4. Collection of Feces, Blood, Organs, and Tissues

The feces of the rats in the HFD and HFD + APNO groups were collected from the collection boxes during the last three days of the experiment (61–63 days), weighed, and their moisture and lipids contents determined by the Standard AOAC [41].

At the end of the experiment, all the rats were fasted for 12 h before the euthanasia. The blood collected was centrifuged at 3000 rpm for 10 min to obtain the serum, which was then stored at −70 °C for the subsequent biochemical analyses.

The spleen, heart, liver, lung, kidney, white adipose tissue deposits (mesenteric, retroperitoneal, epididymal and inguinal subcutaneous), and skeletal muscles (extensor digitorum longus and soleus) were collected and weighed to determine the relative mass, calculated as the ratio of the mass of tissue per organ and per body weight of the animal. The liver fragments were dehydrated for lipid quantification.

#### 3.6.5. Quantification of Lipids in the Liver and Feces

The lipids were extracted by the Soxhlet’s method [41] with some modifications. Each pre-dried sample was ground and weighed (0.5 g). After this, the samples were inserted in cellulose cartridges that were later placed in contact with hexane (P.A.) in the Soxhlet apparatus, and maintained under boiling conditions for 2 h. The lipid content of the sample was determined gravimetrically after the total evaporation of the solvent in an oven with air circulation at 105 °C. The analyses were carried out in triplicate.

#### 3.6.6. Biochemical Analysis

The serum levels of total cholesterol, HDL-cholesterol, triglycerides, aspartate aminotransferase (AST), alanine aminotransferase (ALT), creatinine, and urea were determined in an automated Cobas Integra 400 Plus analyzer (Roche ™, Rotkreuz, Switzerland), using specific Roche® reagents. The LDL-cholesterol was calculated by the Friedwald formula [44]:

LDL cholesterol = total cholesterol − HDL cholesterol − Triglycerides/5.

### 3.7. Statistical Analysis

The results were expressed as the mean ± standard error of the mean (SEM). The one-way analysis of variance (ANOVA) and Student Newman–Keuls post-test were used to compare the results of the different experimental groups. The *t*-test was used for the comparison between the two groups. The results were considered significant when *P* < 0.05. The statistical analyses were carried out using the GraphPad Prism 5.0 software (San Diego, CA, USA).

## 4. Conclusions

This study showed that the *A. phalerata* nut oil administrated in hyperlipidemic rats induced by a high-fructose diet led to a reduction in body mass and in the mesenteric white adipose tissue, as well as having a hypocholesterolemic effect, possibly mediated by the increased body temperature and elimination of water and lipids in the feces. The beneficial effects were probably due to the action of the oleic, lauric, and linoleic fatty acids, which are the main fatty acids in APNO. Thus, the *A. phalerata* nut oil has a potential use as a functional food and nutritional supplement for the prevention and treatment of hypercholesterolemia and metabolic alterations related to obesity.

## Figures and Tables

**Table 1:** Fatty acid composition of *A. phalerata* nut oil (APNO).

Fatty Acid	APNO (g·100 g^−1^ of Total Fatty Acid)
SAFA	
Caproic acid (C 6:0)	0.27
Caprylic acid (C 8:0)	3.84
Capric acid (C 10:0)	4.13
Lauric acid (C 12:0)	28.87
Myristic acid (C 14:0)	12.00
Pentadecanoic acid (C 15:0)	0.03
Palmitic acid (C 16:0)	10. 70
Margaric acid (C 17:0)	0.04
Stearic acid (C18:0)	4.24
Eicosanoic acid (C 20:0)	0.10
Docosanoic acid (C 22:0)	0.02
Lignoceric acid (C 24:0)	0.07
**Total SAFA**	**64.31**
MUFA	
Palmitoleic acid (C16:1 n-7)	0.06
cis-10-heptadecenoic acid (C 17:1)	0.03
Oleic acid (C18:1 n-9)	30.70
Eicosenoic acid (C 20:1 n-9)	0.11
**Total MUFA**	**30.90**
PUFA	
Linoleic acid (C 18:2 n-6)	4.75
α-Linolenic (C 18:3 n-3)	0.04
**Total PUFA**	**4.79**
Total fatty acid	100

SAFA = saturated fatty acid; MUFA = monounsaturated fatty acid; PUFA = polyunsaturated fatty acid.

**Table 2:** The daily mean by animal of food (g) and water (mL) ingestion, and the relative mass of white adipose tissue depots, skeletal muscle, and organs of normolipidemic rats (Control) and high-fructose diet-induced hyperlipidemic rats treated with water (HFD) and *A. phalerata* nut oil (1.2 mL·Kg^−1^ of body weight, HFD + APNO) for 63 days.

Parameters	Groups		
	Control (*n* = 5)	HFD (*n* = 8)	HFD + APNO (*n* = 8)
**Daily mean by animal**			
Food intake (g/day)	23.40 ± 0.70 ^a^	26.74 ± 1.07 ^b^	25.30 ± 1.03 ^ab^
Water intake (mL/day)	47.97 ± 1.32 ^a^	36.71 ± 0.82 ^b^	31.06 ± 0.61 ^c^
**White adipose tissue** (g·100 g^–1^ BW)			
Mesenteric	0.80 ± 0.05 ^a^	0.96 ± 0.09 ^ab^	0.72 ± 0.04 ^ac^
Retroperitoneal	2.27 ± 0.22 ^a^	1.92 ± 0.10 ^a^	1.97 ± 0.19 ^a^
Epididymal	1.77 ± 0.14 ^a^	1.17 ± 0.15 ^b^	1.32 ± 0.10 ^b^
Inguinal subcutaneous	0.14 ± 0.03 ^a^	0.28 ± 0.01 ^b^	0.28 ± 0.03 ^b^
**Skeletal muscle** (mg·g^–1^ BW)			
EDL	0.014 ± 0.001 ^a^	0.014 ± 0.001 ^a^	0.016 ± 0.002 ^a^
Soleus	0.033 ± 0.002 ^a^	0.028 ± 0.002 ^a^	0.029 ± 0.001 ^a^
**Organs** (g·100 g^–1^ BW)			
Spleen	0.117 ± 0.040 ^a^	0.115 ± 0.002 ^a^	0.105 ± 0.003 ^a^
Heart	0.321 ± 0.013 ^a^	0.347 ± 0.010 ^ab^	0.320 ± 0.006 ^ac^
Liver	2.860 ± 0.045 ^a^	2.720 ± 0.054 ^a^	3.230 ± 0.135 ^b^
Lung	0.522 ± 0.068 ^a^	0.486 ± 0.027 ^a^	0.436 ± 0.023 ^a^
Kidney	0.601 ± 0.026 ^a^	0.545 ± 0.018 ^ab^	0.602 ± 0.014 ^ac^
**Lipids (%)**			
Liver	13.92 ± 0.76 ^a^	8.83 ± 0.79 ^b^	17.04 ± 1.70 ^a^

EDL = extensor digitorum longus. Results are expressed as the mean ± SEM. The data were analyzed by One-way ANOVA test followed by Student Newman–Keuls post-test. Different superscript letters between columns indicate significant differences at *P* < 0.05.

**Table 3:** The metabolic parameters of high-fructose diet-induced hyperlipidemic rats treated with water (HFD) or *A. phalerata* nut oil (HFD + APNO) made available for 24 h in metabolic cages.

Parameters	Experimental Groups	
	HFD (*n* = 8)	HFD + APNO (*n* = 8)
Food intake (g)	17.90 ± 1.38	19.86 ± 1.55
Water intake (mL)	17.93 ± 1.91	14.88 ± 0.85
Urinary excretion (mL)	5.00 ± 1.13	1.64 ± 0.34 **
Feces (g)	4.72 ± 0.50	4.69 ± 0.51
Humidity of feces (%)	24.24 ± 0.13	48.93 ± 0.23 ***
Dry feces (%)	75.75 ± 0.12	51.06 ± 0.23 ***
Lipid of feces (%)	2.26 ± 0.07	2.99 ± 0.11 *
Anal temperature (° C)	35.67 ± 0.55	36.95 ± 0.24 *

Results are expressed as mean ± SEM. HFD versus HFD + APNO. * *P* < 0.05, ** *P* < 0.01, and *** *P <* 0.001. Results are expressed as mean ± SEM. The data was analyzed by Unpaired *t*-test non-parametric tests. HFD versus HFD + APNO. * *P* < 0.05, ** *P* < 0.01, and *** *P <* 0.001.

**Table 4:** The biochemical, hepatic, and renal parameters of normolipidemic rats (Control) and high-fructose-diet-induced hyperlipidemic rats treated with water (HFD), ciprofibrate (2 mg·Kg^−1^ of body weight, HFD + C), simvastatin (30 mg·Kg^−1^ of body weight, HFD + S) and *A. phalerata* nut oil (1.2 mL·Kg^−1^ of body weight, HFD + APNO) for 63 days.

Parameters	Groups				
	Control (*n* = 5)	HFD (*n* = 8)	HFD + C (*n* = 7)	HFD + S (*n* = 6)	HFD + APNO (*n* = 8)
TG (mg·dL^−1^)	113.0 ± 5.2 ^a^	241.2 ± 28.1 ^b^	155.1 ± 14.2 ^a^	118.9 ± 7.0 ^a^	243.0 ± 20.0 ^b^
TC (mg·dL^−1^)	79.0 ± 2.01 ^a^	117.2 ± 12.9 ^b^	84.4 ± 4.3 ^a^	90.3 ± 5.2 ^a^	93.3 ± 2.5 ^a^
HDL (mg·dL^−1^)	45.08 ± 2.44 ^a^	54.59 ± 3.79 ^a^	47.54 ± 2.35 ^a^	54.11 ± 3.11 ^a^	49.07 ± 1.73 ^a^
LDL (mg·dL^−1^)	11.32 ± 2.40 ^a^	19.73 ± 3.19^a^	8.74 ± 2.83 ^a^	17.25 ± 5.18 ^a^	13.94 ± 7.07 ^a^
ALT (U·L^−1^)	53.2 ± 3.3 ^a^	65.4 ± 7.0 ^a^	65.0 ± 4.1 ^a^	64.8 ± 5.7 ^a^	66.1 ± 7.5 ^a^
AST (U·L^−1^)	174.0 ± 6.5 ^a^	178.0 ± 14.5 ^a^	195.0 ± 16.0 ^a^	219.5 ± 19.7 ^a^	170.8 ± 11.9 ^a^
CR (U·L^−1^)	0.3 ± 0.0 ^a^	0.3 ± 0.0 ^a^	0.3 ± 0.0 ^a^	0.3 ± 0.0 ^a^	0.3 ± 0.0 ^a^
UREA	22.9 ± 1.9 ^a^	30.0 ± 3.0 ^a^	25.9 ± 3.7 ^a^	20.9 ± 1.4 ^a^	19.8 ± 1.9 ^a^

TG = serum triglycerides; TC = serum total cholesterol; ALT = alanine aminotransferase; AST = aspartate aminotransferase; CR = creatinine. Results are expressed as the mean ± SEM. The data was analyzed by one-way ANOVA test followed by Student Newman–Keuls post-test. Different superscript letters between columns indicate significant differences at *P* < 0.05.

**Table 5:** The proximal composition of diets.

Diet	Humidity (%)	Lipids (%)	Ashes (%)	Proteins (%)	Carbohydrate (%)	Energy (Kcal∙100g^−1^)
CD	10.70 ± 0.04	1.50 ± 0.12	8.21 ± 0.14	26.14 ± 0.95	59.40 ± 0.64	332.51 ± 0.46
HFD	3.98 ± 0.12	0.35 ± 0.00	3.04 ± 0.08	14.28 ± 1.80	84.39 ± 0.43	374.26 ± 0.64

CD = standard chow diet; HFD = high-fructose diet.
